# Risk of death from cardiovascular disease following breast cancer in Southeast Asia: a prospective cohort study

**DOI:** 10.1038/s41598-017-01540-7

**Published:** 2017-05-02

**Authors:** S. A. M. Gernaat, P. J. Ho, N. Rijnberg, S. C. Lee, S. H. Lim, Y. S. Yap, D. E. Grobbee, M. Hartman, H. M. Verkooijen

**Affiliations:** 1 University Medical Center Utrecht, Julius Center for Health Sciences and Primary Care, Department of Epidemiology, Utrecht, 3584 CX The Netherlands; 2 National University of Singapore, Saw Swee Hock School of Public Health, Singapore, 117549 Singapore; 3 Academic Medical Center, Division of Internal Medicine, Amsterdam, 1105 AZ The Netherlands; 4 National University Cancer Institute, National University Health System, Department of Hematology-oncology, Singapore, 119074 Singapore; 5 KK Women’s and Children’s Hospital, KK Breast Department, Singapore, 229899 Singapore; 6 National Cancer Centre Singapore, Division of Medical Oncology, Singapore, 169610 Singapore; 7 National University Hospital Singapore, Department of Surgery, Singapore, 119074 Singapore; 8 University Medical Center Utrecht, Imaging Division, Utrecht, 3584 CX The Netherlands

## Abstract

Breast cancer incidence and survival is high in Southeast Asia. As such, many women diagnosed with breast cancer are at risk of dying of other causes. Given the increased risk of cardiotoxicity induced by breast cancer treatments, it is important to identify patients at high risk of cardiovascular disease (CVD) mortality. The aim of this study was to investigate if this risk varies by age and ethnicity. Patient details were obtained from 5,868 Chinese, Malay, and Indian women diagnosed with *in situ* or non-metastasized invasive breast cancer at the National University Hospital of Singapore and KK Women’s and Children’s Hospital in Singapore. Death causes were obtained from the National Registry of Births and Deaths. Flexible parametric survival models estimated CVD mortality rates and hazard ratios. During a median follow-up of six years, 1,010 deaths occurred of which 6.8% were due to CVD. CVD mortality rates of older women peaked within the first year following diagnosis and increased over time since diagnosis. Indian had more than double the risk of CVD mortality than Chinese, independent of age at diagnosis and stage. Taking ethnicity and age into account may promote CVD risk stratification and management in (Southeast Asian) women with breast cancer.

## Introduction

Breast cancer incidence is rising dramatically in Southeast Asia, which, in combination with improved survival rates due to earlier detection and improved treatments, leads to an increasing number of women living with or after breast cancer^1, 2^. Many of these women are at risk of dying of other causes than breast cancer^3–6^. Breast cancer patients treated with adjuvant treatments such as radiotherapy or chemotherapy may be at increased absolute risk of treatment-induced cardiac toxicity, and therefore, to develop CVD^7–10^. As such, CVD is now an important cause of death among women with breast cancer, with up to 10% of breast cancer patients dying of CVD^4–6^.

In the multi-ethnic population of Southeast Asia, comprising mainly of Chinese, Malay and Indian, there are ethnic differences in breast cancer survival^11^. Malay breast cancer patients have a higher risk of overall death following breast cancer compared to Chinese, regardless of age at diagnosis, tumor and treatment characteristics^11^. Furthermore, ethnic differences in severity of and death due to CVD is observed in high risk Southeast Asian patients, though not in breast cancer patients, with lowest severity of CVD in Chinese and highest CVD-specific mortality rates in Malay^12, 13^.

Given the increased risk of cardiac toxicity induced by breast cancer treatments, it is important to identify patients at high risk of CVD mortality. Taking the risk (factors) of CVD into account with breast cancer treatment decision will promote personalized CVD risk stratification and management of (Southeast Asian) breast cancer patients.

The objective of the present study was to investigate the risk of CVD mortality following breast cancer in Southeast Asia, and to assess if these risks vary by age at diagnosis and ethnicity.

## Methods and Materials

### Cohort study

This prospective cohort study was conducted within the context of two hospital-based breast cancer registries from the National University Hospital (NUH) in Singapore and KK Women’s and Children’s Hospital (KKH) in Singapore. All methods were carried out in accordance with the guidelines and regulations of NUH and KKH. The NUH received ethic approval from the National Healthcare Group Domain Specific Review Board and KKH received approval from SingHealth Centralized Institutional Review Board. The need for informed consent from all patients was waived by these ethics review boards.

The NUH breast cancer registry contains data of all 4,122 women diagnosed with breast cancer between 1990 and 2011. Details of the registry have been described elsewhere^14^. Data were collected retrospectively for 492 patients diagnosed between 1990 and 1995, and prospectively for 3,630 patients diagnosed after 1995. The KKH breast cancer registry contains prospectively collected data of all 2,192 women diagnosed with breast cancer between 2005 and 2015. Both registries from NUH and KKH include data on patient’s socio-demographic characteristics, tumor and treatment profile.

For the present study, breast cancer patients with distant metastases at diagnosis (n = 431) were excluded, leaving 5,868 women with *in situ* or non-metastasized invasive breast cancer. Ethnicity was categorized into four groups: Chinese, Malay, Indian and other (e.g. Eurasian, Caucasian). Other variables of interest included age at diagnosis, year of diagnosis, tumor stage at diagnosis according to the classification of malignant tumors (TNM)^15^, tumor differentiation (good, moderate, poor, unknown), estrogen receptor status (positive >1% of tumor cells expressing estrogen receptors, negative, unknown), breast cancer treatment including surgery (yes, no, unknown), chemotherapy (yes, no, unknown), radiotherapy (yes, no, unknown) and hormonal therapy (yes, no, unknown).

### Outcomes

For the NUH breast cancer registry, information on vital status and cause of death was obtained from the National Registry of Births and Deaths in Singapore on April 30, 2015, and was complete for all 3,841 breast cancer patients from NUH. For the KKH breast cancer registry, vital status of each patient was known until last clinical follow-up visit, and cause of death was verified from the National Registry of Births and Deaths in Singapore for patients who did not show up at the follow-up visit and could not be contacted. The National Registry of Births and Deaths has certificates on causes of death issued by doctors or authorized medical practitioners within 24 hours after death. Causes of death were classified according to the International Classification of Diseases (ICD) versions 8, 9 or 10 codes, and regrouped into: death as a result of CVD (ICD8: 390 to 459; ICD9: 390 to 459; ICD10: 100 to 199), death as a result of breast cancer (ICD8: 174; ICD9: 174; ICD10: C50), and death as a result of all other cause (all ICD codes except those already listed).

### Statistical analysis

Demographics, tumor characteristics, treatment details and survival time were described for the total population and for the total number of deaths, deaths from CVD, deaths from breast cancer and deaths from all other cause within 10 years of diagnosis. Time at risk for specific causes of death (i.e. CVD, breast cancer, all other cause) was calculated as the minimum of time between date of breast cancer diagnosis and date of death, end of study (April 30, 2015), last clinical follow-up visit, or ten years post diagnosis, whichever occurred first.

Flexible parametric survival models were used to estimate both mortality rates and hazard ratios (HRs) for death from CVD, death from breast cancer, and death from all other cause within 10 years after diagnosis, using restricted cubic spline functions^16^. First, we estimated unadjusted mortality rates for death from CVD, breast cancer and all other cause within 10 years after diagnosis for age at diagnosis in three categories (<50, 50–69, ≥70 years). This model used three internal knots in total: two internal knots for the association between age at diagnosis in categories and follow-up time, and one knot for the time varying effect of age. Mortality rates were reported per 1,000 person-years using days as underlying time.

Second, (crude) HRs with 95% confidence intervals (CI) were estimated as a measure of association between the main determinant ethnicity – Chinese, Malay, and Indian; other ethnicities were excluded due to the small number of patients with heterogeneous origins (n = 104) – and outcomes (i.e. death from CVD, breast cancer, all other cause). These HRs are similar to those estimated by the Cox’s proportional hazard models. Flexible parametric survival models, however, have the ability to estimate the baseline mortality rates allowing the HRs to change over time. Crude HRs for determinants other than ethnicity and age at diagnosis were estimated in a similar manner. Those that were statistically significant were included in the multivariable model. Due to having only 67 CVD-specific deaths, the maximum degrees of freedom used was limited to six for CVD-specific mortality. The following models were used for the analysis: (1) CVD-specific mortality model which has zero internal knots assuming proportional hazards over 10 years of follow-up and is adjusted for age at diagnosis (per 10 year increase) and stage (*in situ* to stage II, stage III or unknown, women with unknown stage showed similar mortality risks as those with stage III), (2) breast cancer-specific mortality model which has two internal knots and is adjusted for age at diagnosis (per 10 year increase), categorized year of diagnosis (1990–2005, 2006–2010, 2011–2015), stage (*in situ* to stage II, stage III or unknown), tumor differentiation grade, chemotherapy, radiotherapy, and hormonal therapy, and (3) the model for all other cause-specific mortality which has zero internal knots and is adjusted for age at diagnosis (per 10 year increase), categorized TNM stage (*in situ* to stage II, stage III or unknown), and radiotherapy.

## Results

Of the 5,868 women with breast cancer in the study, median age at diagnosis was 52 years (interquartile range: 45–60) and median follow-up was six years (interquartile range: 3–10) (Table 1). Of these women, 79% (n = 4,663) were Chinese, 12% (n = 694) were Malay, 6% (n = 351) were Indian, and 3% (n = 160) were women with another ethnicity. The majority of women (86%, n = 5,024) were diagnosed with breast cancer after 1999. Thirteen percent of women (n = 764) were diagnosed with carcinoma *in situ*, 24% (n = 1,381) with stage I, 37% (n = 2,181) with stage II, 13% (n = 756) with stage III, and stage was unknown for 13% (n = 786).

**Table 1:** Characteristics of the total study population and by death from cardiovascular disease, breast cancer, and all other cause, within 10 years of diagnosis in Southeast Asian women with breast cancer.

	Total study population	Total deaths	Deaths from cardiovascular disease	Deaths from breast cancer	Deaths from all other cause
	n = 5,868	n = 1,010	n = 67 (6.8%)	n = 774 (76.6%)	n = 169 (16.7%)
**Ethnicity, n (%)**					
Chinese	4,663 (79.5)	740 (73.3)	50 (6.8)	555 (75.0)	135 (18.2)
Malay	694 (11.9)	178 (17.6)	8 (4.4)	153 (86.0)	17 (9.6)
Indian	351 (6.0)	61 (6.0)	8 (13.1)	47 (77.0)	6 (9.9)
Other*	160 (2.6)	31 (3.1)	1 (3.0)	19 (61.0)	11 (35.0)
Median (IQR) age at diagnosis, years	52 (45–60)	56 (47–68)	69 (58–77)	53 (45–63)	65 (54–74)
**Age at diagnosis in groups, n (%)**					
<50	2530 (43.1)	346 (34.3)	9 (2.6)	312 (90.2)	25 (7.2)
50–69	2781 (47.4)	460 (45.5)	25 (5.4)	347 (75.4)	88 (19.1)
≥65	940 (16.0)	286 (28.0)	37 (55.0)	164 (21.0)	85 (50.0)
Unknown	9 (0.0)	4 (0.0)	0 (0.0)	4 (1.0)	0 (0.0)
Median (IQR) survival time, years	6 (3–10)	3 (2–5)	4 (2–7)	3 (2–5)	3 (2–6)
**Calendar time at diagnosis, n (%)**					
1990–1994	380 (6.5)	121 (11.9)	9 (7.4)	101 (83.5)	11 (9.1)
1995–1999	445 (7.6)	119 (11.8)	5 (4.2)	97 (81.5)	17 (14.3)
2000–2006	1,913 (32.6)	447 (44.3)	31 (6.9)	350 (78.3)	66 (14.7)
≥2007	3,111 (53.0)	317 (31.4)	22 (6.9)	222 (29.0)	73 (43.0)
Unknown	19 (0.0)	6 (1.0)	0 (0.0)	4 (1.0)	2 (1.0)
**TNM stage, n (%)**					
*In situ*	764 (13.0)	17 (1.7)	3 (17.6)	5 (29.4)	9 (52.9)
I	1,381 (23.5)	91 (9.0)	9 (9.9)	49 (53.8)	33 (36.3)
II	2,181 (37.2)	328 (32.5)	27 (8.2)	253 (77.1)	48 (14.6)
III	756 (12.9)	261 (25.8)	9 (3.4)	226 (86.6)	26 (10.0)
Unknown	786 (13.4)	313 (31.0)	19 (6.1)	241 (77.0)	53 (16.9)
**Tumor differentiation grade, n (%)**					
Good	713 (12.2)	56 (5.5)	10 (17.8)	23 (41.1)	23 (41.1)
Moderate	1,998 (34.0)	286 (58.0)	26 (9.1)	206 (72.0)	54 (18.9)
Poor	2,158 (36.8)	475 (47.0)	20 (4.2)	394 (82.9)	61 (12.8)
Unknown	999 (17.0)	193 (19.1)	11 (5.7)	151 (78.2)	31 (16.1)
**Estrogen receptor status, n (%)**					
Positive	1,772 (30.2)	376 (37.2)	37 (9.8)	269 (71.5)	70 (18.6)
Negative	2,537 (43.2)	382 (37.8)	17 (4.4)	314 (82.2)	51 (13.4)
Unknown	1,559 (26.6)	252 (25.0)	13 (5.2)	191 (75.8)	48 (19.0)
**Surgery, n (%)**					
Yes	5,262 (89.7)	758 (75.0)	52 (6.9)	579 (76.4)	127 (16.8)
No	345 (5.9)	218 (21.6)	14 (6.4)	166 (76.1)	38 (17.5)
Unknown	261 (4.4)	34 (3.4)	1 (2.9)	29 (85.3)	4 (11.8)
**Chemotherapy, n (%)**					
Yes	2,797 (47.7)	519 (51.3)	14 (2.7)	450 (86.7)	55 (10.6)
No	2,671 (45.5)	453 (44.9)	52 (11.5)	293 (64.7)	108 (23.8)
Unknown	400 (6.8)	38 (3.8)	1 (2.6)	31 (81.6)	6 (15.8)
**Radiotherapy, n (%)**					
Yes	2,735 (46.6)	462 (45.7)	20 (4.3)	383 (82.9)	59 (12.8)
No	2,637 (44.9)	506 (50.1)	45 (8.9)	358 (70.8)	103 (20.4)
Unknown	496 (8.5)	42 (4.2)	2 (4.8)	33 (78.6)	7 (16.7)
**Hormonal therapy, n (%)**					
Yes	3,355 (57.2)	537 (53.2)	44 (8.2)	393 (73.2)	100 (18.6)
No	2,160 (36.8)	433 (42.9)	22 (5.1)	348 (80.4)	63 (14.5)
Unknown	353 (6.0)	40 (4.0)	1 (2.5)	33 (82.5)	6 (15.0)

Abbreviations: IQR, Interquartile range; TNM, Classification of Malignant Tumors. *Includes e.g. Sikh, Eurasian and Caucasian. Column percentages presented for total study population and total deaths; Row percentages (percentages of total deaths) presented for deaths from cardiovascular disease, deaths from breast cancer, and deaths from all other cause.

In total, 1,010 deaths occurred within 10 years of follow-up of which 6.8% (n = 67) were due to CVD (Table 1). Of these, 24 deaths were due to acute myocardial infarction, 4 deaths were due to congestive heart failure, 16 deaths were due to acute ischemic heart disease, 3 deaths were due to coronary artery disease, 11 deaths were due to cerebrovascular death (anoxic brain damage), and 9 deaths were due to other heart disease problems (cardiorespiratory failure) (data not presented). The most common cause of death was breast cancer accounting for 76.6% (n = 774) of all deaths, and in 16.7% (n = 169), death was due to other causes than breast cancer or CVD. Of the 740 Chinese women with breast cancer who died, 6.8% (n = 50) died of CVD, 75.0% (n = 555) died of breast cancer, and 18.2% (n = 135) died of other causes. Of the 178 Malay women with breast cancer who died, 4.4% (n = 8) died of CVD, 86.0% (n = 153) died of breast cancer, and 9.6% (n = 47) died of other causes. Furthermore, of the 61 Indian women with breast cancer who died, 13.1% (n = 8) died of CVD, 77.0% (n = 47) died of breast cancer, and 9.9% (n = 6) died of other causes.

**Figure 1:**
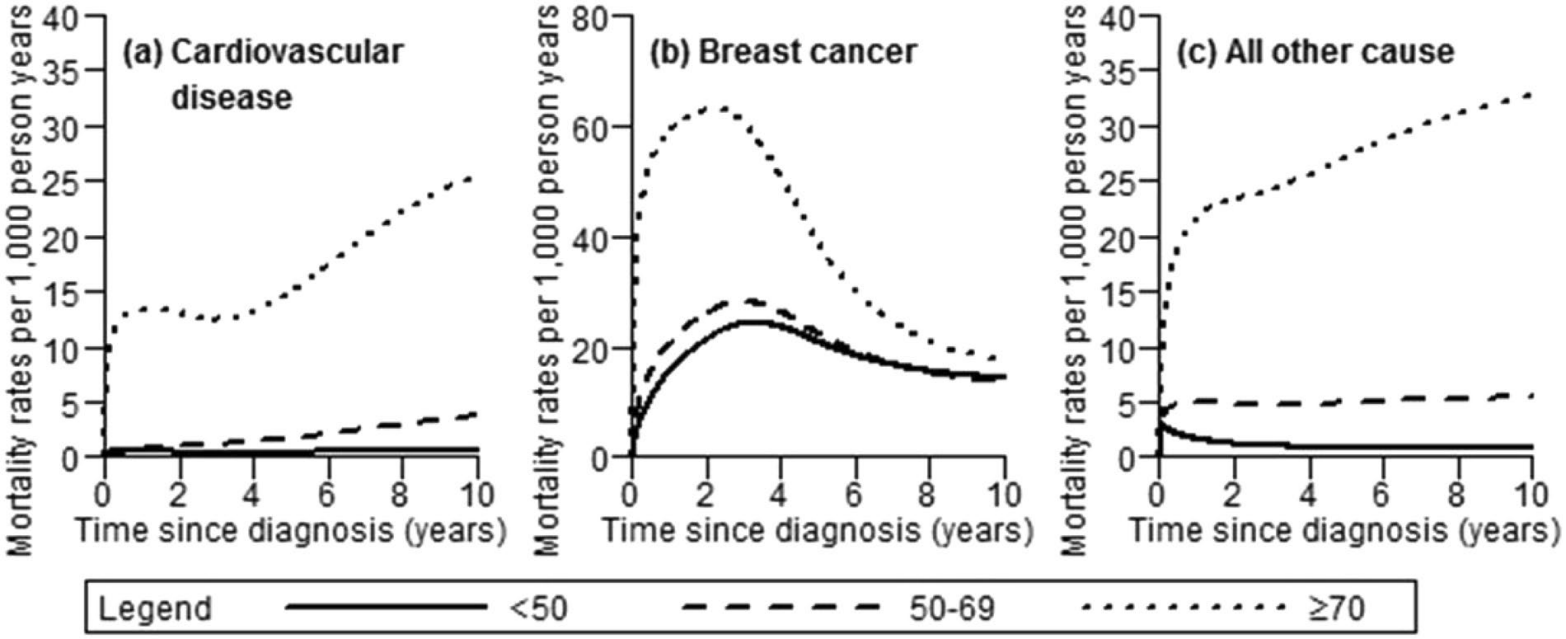
Estimated mortality rates within 10 years of diagnosis in Southeast Asian women with breast cancer diagnosed between 1990 and 2011 (mortality rates estimated from flexible parametric survival models with age as only covariate, allowing age effect to vary by time since diagnosis). Death due to (**a**) cardiovascular disease, (**b**) breast cancer, (**c**) all other cause.

Among women over 70 years at breast cancer diagnosis, CVD mortality rates peaked in the first year after diagnosis followed by a decrease until approximately three years after breast cancer diagnosis (Fig. 1). Thereafter, CVD mortality rates rose until approximately 12 per 1,000 person-years after 10 years of follow-up. CVD mortality rates among women aged 50 to 69 years at breast cancer diagnosis gradually increased with follow-up until approximately 4 per 1,000 person-years after 10 years of follow-up. In women younger than 50 years, CVD mortality remained almost constant at less than 1 per 1,000 person years during the 10 years of follow-up. Breast cancer-specific mortality rates peaked at one to three years after diagnoses for all breast cancer patients. Women over 70 years had substantially higher breast cancer specific mortality rates within the first five years after diagnosis than younger women. After eight years since diagnosis, similar breast cancer-specific mortality rates (approximately 20 per 1,000 person-years) were seen in all age groups. Mortality rates from other causes were highest among women aged over 70 years, and showed an early peak within the first year after breast cancer diagnosis followed by a steady increase over time until approximately 25 per 1,000 person-years after ten years of follow-up. In the younger age groups, mortality rates from other causes remained stable over follow-up time at approximately five per 1,000 person-years for women aged between 50 and 69 years and less than one per 1,000 person-years for women younger than 50 years.

The risk of death from CVD within 10 years of diagnosis in Indian women was more than double the risk of Chinese women (HR = 2.5, 95% CI = 1.2–5.2), independent of age at diagnosis and stage (Table 2). Furthermore, the risk of death from breast cancer in Malay women was almost double the risk of Chinese women (HR = 1.9, 95% CI = 1.6–2.3) independent of age at diagnosis, tumor stage, tumor differentiation grade, chemotherapy, radiotherapy, and hormonal therapy.

**Table 2:** Crude and adjusted hazard ratios of death from cardiovascular disease, breast cancer, and all other cause within 10 years of diagnosis in Southeast Asian women with breast cancer.

	Cardiovascular disease				Breast cancer				All other cause			
	Crude		Adjusted		Crude		Adjusted		Crude		Adjusted	
	HR	95% CI	HR	95% CI	HR	95% CI	HR	95% CI	HR	95% CI	HR	95% CI
**Ethnicity**												
Chinese	1.0	—	1.0	—	1.0	—	1.0	—	1.0	—	1.0	—
Malay	1.2	0.6 to 2.6	1.8	0.9 to 3.9	2.1	1.8 to 2.6	1.9	1.6 to 2.3	1.0	0.6 to 1.6	1.2	0.7 to 2.0
Indian	2.2	1.0 to 4.6	2.5	1.2 to 5.2	1.2	0.9 to 1.6	1.2	0.9 to 1.6	0.6	0.3 to 1.4	0.6	0.3 to 1.4
Age at diagnosis*	2.8	2.4 to 3.5	2.7	2.2 to 3.4	1.2	1.2 to 1.3	1.3	1.2 to 1.4	2.3	2.0 to 2.6	2.0	1.8 to 2.3
**TNM stage**												
*In situ*	0.4	0.1 to 1.3	0.5	0.2 to 1.6	0.1	0.0 to 0.2	0.1	0.0 to 0.2	0.5	0.2 to 1.0	0.6	0.3 to 1.2
1–2	1.0	—	1.0	—	1.0	—	1.0	—	1.0	—	1.0	—
3	1.3	0.6 to 2.8	1.2	0.6 to 2.7	4.5	3.7 to 5.3	3.6	3.0 to 4.4	1.8	1.1 to 2.8	1.9	1.1 to 3.1
unknown	3.0	1.7 to 5.2	1.7	1.0 to 3.1	4.5	3.8 to 5.4	6.0	4.9 to 7.3	3.3	2.3 to 4.8	2.7	1.8 to 4.0
**Year of diagnosis**												
2011–2015	1.0	—	—		1.0	—	1.0	—	1.0	—	—	
2006–2010	3.0	0.7 to 12.8			1.7	1.2 to 2.5	1.6	1.1 to 2.3	1.1	0.6 to 1.9		
1990–2005	2.7	0.6 to 11.7			2.1	1.5 to 3.0	2.1	1.5 to 3.1	0.8	0.4 to 1.4		
**Tumor differentiation grade**												
Good	1.0	—	—		1.0	—	1.0	—	1.0	—	—	
Moderate	1.0	0.5 to 2.0			3.3	2.1 to 5.0	3.2	2.0 to 4.9	0.8	0.5 to 1.4		
Poor	0.8	0.4 to 1.6			6.6	4.3 to 10.0	5.5	3.6 to 8.4	1.0	0.6 to 1.7		
Unknown	1.0	0.4 to 2.3			5.5	3.5 to 8.5	4.8	3.0 to 7.6	1.0	0.6 to 1.8		
**Estrogen receptor status**												
Positive	2.1	1.2 to 3.7	—		0.9	0.7 to 1.0	—		1.4	1.0 to 2.1	—	
Negative	1.0	—			1.0	—			1.0	—		
Unknown	1.0	0.5 to 2.1			0.8	0.7 to 1.0			1.2	0.8 to 1.8		
**Chemotherapy**												
Yes	0.3	0.2 to 0.5	—		1.6	1.4 to 1.8	1.4	1.1 to 1.6	0.5	0.4 to 0.7	—	
No	1.0	—			1.0	—	1.0	—	1.0	—		
Unknown	0.2	0.0 to 1.1			0.8	0.6 to 1.2	0.6	0.2 to 2.0	0.5	0.2 to 1.1		
**Radiotherapy**												
Yes	0.4	0.2 to 0.7	—		1.0	0.9 to 1.2	0.9	0.7 to 1.0	0.5	0.4 to 0.8	0.8	0.5 to 1.1
No	1.0	—			1.0	—	1.0	—	1.0	—	1.0	—
Unknown	0.3	0.1 to 1.2			0.6	0.4 to 0.9	0.5	0.2 to 1.2	0.5	0.2 to 1.0	0.4	0.2 to 0.8
**Hormonal therapy**												
Yes	1.2	0.7 to 2.1	—		0.7	0.6 to 0.8	0.7	0.6 to 0.8	1.1	0.8 to 1.5	—	
No	1.0	—			1.0	—	1.0	—	1.0	—		
Unknown	0.3	0.0 to 2.2			0.6	0.4 to 0.8	0.6	0.2 to 1.7	0.7	0.3 to 1.6		

Abbreviations: HR, hazard ratio; CI, confidence interval; TNM, Classification of Malignant Tumors. *Per 10 year increase. The HRs are estimated with a flexible parametric survival model including two internal knots for death from breast cancer and zero internal knots for death from cardiovascular disease and all other cause (i.e., assuming proportional hazards over 10 years of follow-up). Adjusted HRs for death from CVD by ethnicity are adjusted for age at diagnosis (per ten year increase) and TNM stage. Adjusted HRs for death from breast cancer by ethnicity are adjusted for age at diagnosis (per ten year increase), TNM stage, tumor differentiation grade, chemotherapy, radiotherapy, and hormonal therapy. Adjusted HRs for death from all other cause by ethnicity are adjusted for age at diagnosis (per ten year increase), TNM stage, and radiotherapy.

## Discussion

Breast cancer survival is improving in Southeast Asia. As such, the number of breast cancer survivors at risk of dying of other causes is increasing. Given the increased risk of cardiac toxicity induced by breast cancer treatments, it is important to identify patients at high risk of CVD mortality.

The present study shows that the risk of CVD mortality following breast cancer in multi-ethnic Southeast Asia is generally low during the first decade after diagnosis. Breast cancer is still the main cause of death. The risk of CVD mortality is increased in women with higher age at breast cancer diagnosis and in Indian women.

Among women with a higher age at breast cancer diagnosis, CVD mortality rates peak within the first year following diagnosis and show an overall increase during the ten years of follow-up. Similar results have been reported among Caucasian women with breast cancer^4^. Our unadjusted survival analysis show that women who have been treated with chemotherapy or radiotherapy have a lower risk of dying of CVD than women who did not receive those therapies. Moreover, women with a positive estrogen receptor status have a higher risk of dying of CVD than women with a negative receptor status. A possible explanation is the phenomenon is that women with breast cancer who receive chemotherapy, radiotherapy, and/or have a negative estrogen receptor status have a higher TNM stage and therefore die more often due to breast cancer than due to CVD. Also, some selection may have taken place, as women who are at increased risk of CVD are less likely to be treated with cardiac toxic chemotherapy or (left-sided) radiotherapy. Furthermore, our study shows that Indian women have an increased risk of CVD mortality following breast cancer compared to Chinese women. Variation in the risk of CVD mortality by ethnicity in our population may be due to genetic differences and/or differences in lifestyle associated comorbidities like obesity and diabetes and dietary habits^17, 18^. Differences in the presence of CVD risk factors between Chinese, Malay and Indian in Southeast Asia have been reported^17, 19–22^. Indian women have the highest rate of central obesity and diabetes^21^, while the rate of obesity is highest among Malay followed by Indian and Chinese^19, 20^. These differences, however, are not fully explained by dietary intake^23^. Moreover, Indians have the highest level of lipoprotein a, which is a causal genetic risk factor for cardiovascular disease^22^.

In the current study, CVD mortality rates of women with breast cancer aged over 70 years were over 10 per 1,000 person-years shortly after diagnosis until 10 years after diagnosis, however, CVD mortality rates of women from the general population in Singapore aged over 70 years ranged from 19 per 1,000 person-years in 2005 to 17 per 1,000 person-years in 2015 (data not presented)^24^. Furthermore, in this study, CVD mortality rates of women with breast cancer aged 50 to 69 years were approximately 0.3 per 1,000 person-years throughout the 10 years follow-up while the CVD mortality rates of women from the general population in Singapore aged 50 to 69 years ranged from 1.4 per 1,000 person-years in 2005 to 0.9 per 1,000 person-years in 2015 (data not presented)^24^. These numbers show that mortality rates of CVD among women with breast cancer patients in Singapore are somewhat lower than that of women from the general population in Singapore.

In the present study, CVD mortality rates increase over time while breast cancer mortality rates decrease after three years since diagnosis. Colzani *et al.* found similar results among Caucasian women with breast cancer: CVD-specific mortality rates increase after three years since diagnoses in women aged over 55 years at diagnosis, while breast cancer-specific mortality rates decreased after four years since diagnosis among all ages^4^. These results are not surprising as age is a well-known important risk factor of CVD^25^, and the majority of deaths from breast cancer within the first four years following diagnosis in our prospective cohort were women diagnosed with more severe stages i.e. II and III.

We acknowledge that the present study has limitations. The follow-up time of our study population was relatively short, which explains (part of) the low absolute risk of CVD mortality. Previous research has shown, that the risk of CVD mortality increases up to and beyond 20 years after diagnosis^4, 26, 27^, as age is a well-known CVD risk factor^28^ and cardiac toxicity induced by radiotherapy manifest itself many years following treatment^29, 30^. Furthermore, misclassification of cause of death due to CVD could have occurred, especially in cases of sudden death from CVD outside a hospital, for example at home, were it is difficult to state the proper cause of death by doctors or authorized medical practitioners that are not familiar with this particular women.

In conclusion, the risk of CVD mortality is generally low in the first decade following breast cancer diagnosis in Southeast Asia. Women with higher age at breast cancer diagnosis and Indian women are at increased risk of CVD mortality following breast cancer. The notion that women who survive breast cancer will subsequently be at risk for CVD is important in their management and in addition, taking ethnic-specific risks and age into account, may promote optimal prevention of CVD in (Southeast Asian) women with breast cancer. Future research may assess factors, dependent or independent of breast cancer, explaining the variation in risk of CVD mortality according to ethnicity. Furthermore, breast cancer patients would benefit from a personalized CVD risk prediction short after breast cancer diagnosis so that treatment can be adjusted accordingly and CVD management can be initialized.
